# Results on Varextropy Measure of Random Variables

**DOI:** 10.3390/e23030356

**Published:** 2021-03-17

**Authors:** Nastaran Marzban Vaselabadi, Saeid Tahmasebi, Mohammad Reza Kazemi, Francesco Buono

**Affiliations:** 1 Department of Statistics, Persian Gulf University, Bushehr 7516913817, Iran; nastaran.marzban@gmail.com (N.M.V.); tahmasebi@pgu.ac.ir (S.T.); 2 Department of Statistics, Faculty of Science, Fasa University, Fasa 7461686131, Iran; kazemi@fasau.ac.ir; 3 Dipartimento di Matematica e Applicazioni “Renato Caccioppoli”, Università degli Studi di Napoli Federico II, I-80126 Naples, Italy

**Keywords:** extropy, uncertainty, varextropy, residual lifetime, 62B10, 62G30

## Abstract

In 2015, Lad, Sanfilippo and Agrò proposed an alternative measure of uncertainty dual to the entropy known as extropy. This paper provides some results on a dispersion measure of extropy of random variables which is called varextropy and studies several properties of this concept. Especially, the varextropy measure of residual and past lifetimes, order statistics, record values and proportional hazard rate models are discussed. Moreover, the conditional varextropy is considered and some properties of this measure are studied. Finally, a new stochastic comparison method, named varextropy ordering, is introduced and some of its properties are presented.

## 1. Introduction

In the context of information theory, entropy was first proposed by Clausius. He used the concept of entropy to quantitatively express the second law of thermodynamics, which opened up a new path for the development of thermodynamics [1]. This concept was continued by Shannon [2] and since then it has been used in several fields, such as image and signal processing and economics. Let *X* be an absolutely continuous random variable with probability density function (pdf) f(x); the differential entropy is a measure of uncertainty, and is defined by

$$H\left(X\right)=-\int_{-\infty }^{+\infty }f\left(x\right)\log f\left(x\right)dx,$$

where log(·) stands for the natural logarithm with the convention 0log0=0. Song [3] introduced the concept of varentropy (VE) as an excellent alternative for the kurtosis measure. In fact, the VE can be used to compare the heavy-tailed distributions instead of kurtosis measure. Liu [4] studied some properties of VE under the concept of information volatility. Fradelizi et al. [5] obtained an optimal varentropy bound for log-concave distributions. The varentropy of a random variable *X* is defined as

(1) VH(X)=Var(−logf(X)).

Varentropy measures the variability in the information content of *X*. Recently, Di Crescenzo and Paolillo [6] studied the varentropy for residual lifetime. Maadani et al. [7] introduced a method for calculating this measure for the *i*-th order statistic. An alternative measure of uncertainty, known as extropy, was proposed by Lad et al. [8]. For an absolutely continuous random variable *X* with pdf f(x), the extropy is defined as

$$J\left(X\right)=E\left(-\frac{1}{2}f\left(X\right)\right)=-\frac{1}{2}\int_{-\infty }^{+\infty }{\left[f\left(x\right)\right]}^{2}dx=-\frac{1}{2}\int_{0}^{1}f\left(F^{-1}\left(u\right)\right)du,$$

where $F^{-1}\left(u\right)=\inf\left\{x:F\left(x\right)\geq u\right\}$ is the quantile function of the cumulative distribution function (cdf) *F*. Recently, several authors have paid attention to extropy and its applications. Qiu [9] discussed some characterization results, monotone properties, and lower bounds of extropy of order statistics and record values. Moreover, Qiu and Jia [10] focused on the residual extropy of order statistics. Qiu and Jia [11] explored the extropy estimators with applications in testing uniformity.

In some situations, one may have two random variables with the same extropy; then, this problem leads to the well-known question “Which of the extropies is the most appropriate criterion for measuring the uncertainty?”. For example, the extropy values of standard uniform and an exponential distribution with the parameter 2 are both equal to $-\frac{1}{2}$. This question motivates one to investigate the variance of $-\frac{1}{2}f\left(X\right)$, which is called varextropy. Varextropy measure indicates how the information content is scattered around the extropy. It can be shown that the varextropy of the uniform distribution is zero and for exponential distribution of parameter 2 is $\frac{1}{12}$, so in the uniform distribution, extropy is more appropriate for measuring the uncertainty, because the uniform distribution has the least information volatility. In addition to the varentropy, the use of the varextropy is also required. Some comparative results for these measures varentropy and varextropy are conducted in the next section. One can observe that the new introduced varextropy measure is more flexible than the varentropy, in the sense that the latter is free of the model parameters in some cases.

Aiming to analyze the variability of such information content, in the present paper, an alternative measure analogous to (1) is proposed which can be used for measuring the dispersion of the residual and past lifetimes. On the ground of the above remarks, the motivation of this paper is to investigate the varextropy of random lifetime in reliability theory. Accordingly, this paper is organized as follows. In Section 2, at first, the definition of the varextropy measure is given and some of its properties are investigated. Especially, some of its extensions in residual and past lifetimes, order statistics, record values and proportional hazard rate models are provided and the approximate formula for varextropy using Taylor series is proposed. In Section 3, some results on the conditional varextropy measure are obtained. In Section 4, a new stochastic comparison method, named varextropy ordering is introduced, and some of its properties are presented. Throughout this paper, E[·] denotes expectation and $f^{\prime }$ means the derivative of *f*.

## 2. Varextropy Measure

Hereafter, we introduce a measure of uncertainty which can be used as an alternative measure to Shannon entropy. It is known that Shannon entropy measures the uniformity of *f*, hence this remark motivated us to consider the varextropy measure.

**Definition** **1.** *Let X be an absolutely continuous random variable with cdf F and pdf f. The varextropy can be defined as*

(2) $$VJ\left(X\right):=Var\left[-\frac{1}{2}f\left(X\right)\right]=\frac{1}{4}E\left[f^{2}\left(X\right)\right]-J^{2}\left(X\right).$$

Quantile functions $Q\left(u\right)=F^{-1}\left(u\right),\;\;0\leq u\leq 1,$ are efficient alternatives to the cdf in modelling and analysis of statistical data, see, for instance, ref. [12]. Let U∼U(0,1), the corresponding quantile based varextropy of *X*, be defined as

$$\begin{array}{ccc} VJ(X) & = & \frac{1}{4}\left[E\left[f^{2}\left(Q\left(U\right)\right)\right]-E^{2}\left[f\left(Q\left(U\right)\right)\right]\right]\\ & = & \frac{1}{4}\left[\int_{0}^{1}f^{2}\left(F^{-1}\left(u\right)\right)du-{\left(\int_{0}^{1}f\left(F^{-1}\left(u\right)\right)du\right)}^{2}\right]. \end{array}$$

In the following, a few examples are given to illustrate the varextropy for random variables from some distributions.

**Example** **1.**

*(i)*  *If X is uniformly distributed in [0,a], then VJ(X) = VH(X) = 0. As one can see, conceptually, the varextropy is compatible with varentropy and both take values greater than or equal to zero. So, when both varextropy and varentropy are zero, they represent certain information, that is, the event is certain.*
*(ii)*  *If X follows the Weibull distribution with cdf*
  $$F\left(x\right)=1-e^{-\lambda x^{\alpha }},\;x>0,$$
  *then, a direct computation yields*
  $$\begin{array}{ccc} VJ(X) & = & \frac{\alpha^{2}\lambda^{2}}{4\lambda^{\frac{2(\alpha -1)}{\alpha }}}\left[\frac{\Gamma (\frac{2(\alpha -1)}{\alpha }+1)}{3^{\frac{2(\alpha -1)}{\alpha }+1}}-\frac{\Gamma^{2}\left(\frac{\left(\alpha -1\right)}{\alpha }+1\right)}{2^{\frac{2(\alpha -1)}{\alpha }+2}}\right],\\ VH(X) & = & \frac{\pi^{2}}{6}{\left(1-\frac{1}{\alpha }\right)}^{2}+\frac{2}{\alpha }-1. \end{array}$$
  *In particular, when α=1 one has the exponential distribution with $VJ\left(X\right)=\frac{\lambda^{2}}{48}$ and VH(X)=1.*
*(iii)*  *If X follows a power distribution with parameter α>1, i.e., $f\left(x\right)=\alpha x^{\alpha -1}$, x∈(0,1), then, we have*
  $$\begin{array}{ccc} VJ(X) & = & \frac{\alpha^{3}{\left(\alpha -1\right)}^{2}}{4\left(3\alpha -2\right){\left(2\alpha -1\right)}^{2}},\\ VH(X) & = & {\left(\alpha -1\right)}^{2}\dot{\psi }\left(\alpha \right)-{\left(\alpha -1\right)}^{2}\dot{\psi }\left(\alpha +1\right), \end{array}$$
  *where $\dot{\psi }\left(\cdot \right)$ is the trigamma function.*
*(iv)*  *If X follows a two-parameter exponential distribution with density function*
  f(x)=λexp{−λ(x−μ)},x>μ,
  *then, we have $VJ\left(X\right)=\frac{\lambda^{2}}{48}$ and VH(X)=1. In this case, VH does not depend on the parameters and VJ(X)<VH(X) for $\lambda <4\sqrt{3}$.*
*(v)*  *If X follows the Laplace distribution with density function*
  $$f\left(x\right)=\frac{1}{2\beta }\exp\left\{\frac{-|x|}{\beta }\right\},\;x\in R,$$
  *straightforward computations yield $VJ\left(X\right)=\frac{1}{192\beta^{2}}$ and VH(X)=1. By comparing the varextropy of two-parameter exponential and Laplace distributions with $\beta =\frac{1}{\lambda }$, then varextropy of two-parameter exponential distribution is four times as much as Laplace distribution.*
*(vi)*  *If X is beta-distributed with parameters α and β, then*
  $$\begin{array}{ccc} VJ(X) & = & \frac{B(3(\alpha -1)+1,3(\beta -1)+1)}{4B^{3}\left(\alpha ,\beta \right)}-\frac{B^{2}\left(2\left(\alpha -1\right)+1,2\left(\beta -1\right)+1\right)}{4B^{4}\left(\alpha ,\beta \right)},\\ VH(X) & = & {\left(\alpha -1\right)}^{2}\dot{\psi }\left(\alpha \right)+{\left(\beta -1\right)}^{2}\dot{\psi }\left(\beta \right)-{\left(\alpha -1+\beta -1\right)}^{2}\dot{\psi }\left(\alpha +\beta \right), \end{array}$$
  *where B(·,·) and $\dot{\psi }\left(\cdot \right)$ denote the beta and trigamma functions, respectively.*
*(vii)*  *If $X∼N(\mu ,\sigma^{2})$, then $VJ\left(X\right)=\frac{2-\sqrt{3}}{16\pi \sigma^{2}\sqrt{3}}$ and $VH\left(X\right)=\frac{1}{2}$. In this case, the varextropy depends on the scale parameter $\sigma^{2}$, whereas it is independent on the location parameter μ. From the above examples, it can be seen that the varextropy measure is more flexible than the varentropy, in the sense that the latter is free of the model parameters in some cases.*

In the following, some properties of the varextropy, such as its behaviour for symmetric distributions or how it changes under monotonic transformations, are considered.

**Proposition** **1.** *Suppose X is an absolutely continuous non-negative random variable with mean μ=E(X)<+∞ and pdf $f\left(x\right)=\frac{\bar{F}\left(x\right)}{\mu },\;0<x<+\infty$, where $\bar{F}\left(x\right)=1-F\left(x\right)$ is the survival function of X with cdf F. Then, $VJ\left(X\right)=\frac{1}{48\mu^{2}}$.*

**Proposition** **2.** *Let $\tilde{X}$ be an absolutely continuous random variable with pdf $\tilde{f}\left(x\right)=\frac{xf(x)}{\mu },\;x>0$, where f is a fixed pdf with mean μ=E(X)<+∞. Then, $VJ\left(\tilde{X}\right)=\frac{1}{4\mu^{2}}Var\left[Xf\left(X\right)\right]$.*

**Proposition** **3.** *Let X be a symmetric random variable with respect to the finite mean μ=E(X), i.e., F(x+μ)=1−F(μ−x). Then, VJ(X+μ)=VJ(μ−X).*

**Remark** **1.** *Suppose that X is a continuous random variable with a symmetric density function f with respect to μ=0. Then, VJ(|X|)=4VJ(X)=Var[f(X)]. For instance, if $X∼N(\mu ,\sigma^{2})$, from Example 1, one can get the varextropy for the half-normal distribution.*

**Proposition** **4.** *If Y=h(X) is a strictly monotone function of X, then $VJ\left(Y\right)=\frac{1}{4}Var\left(\frac{f(X)}{h^{\prime }\left(X\right)}\right)$. Note that if Y=aX+b, then $VJ\left(Y\right)=\frac{1}{a^{2}}VJ\left(X\right)$, hence the varextropy is invariant under translations.*

**Remark** **2.** *Let X be a random variable with pdf f and let* Φ *be a convex function. Then the* Φ*-entropy is defined by Beigi and Goahri [13] as follows:*

$$H_{\Phi }\left(f\right)=E\left[\Phi \left(f\right)\right]-\Phi \left(E\left(f\right)\right).$$

*For the choice of $\Phi \left(t\right)=\frac{t^{2}}{4}$, we get $H_{\Phi }\left(f\right)=VJ\left(f\right)$.*

In the following, by using Taylor series, an approximate formula for the varextropy is obtained. For this aim, it is enough to approximate E[f(X)] as follows:

(3) $$\begin{array}{c} E\left[f\left(X\right)\right]\approx E\left[f\left(\mu \right)+f^{\prime }\left(\mu \right)\left(X-\mu \right)+\frac{1}{2}f^{\prime \prime }\left(\mu \right){\left(X-\mu \right)}^{2}\right]=f\left(\mu \right)+\frac{1}{2}f^{\prime \prime }\left(\mu \right)Var\left(X\right). \end{array}$$

**Theorem** **1.** *Let X be a random variable with pdf f and mean μ=E(X)<+∞. Then*

$$VJ\left(X\right)\approx \frac{1}{16}{\left(f^{\prime \prime }\left(\mu \right)\right)}^{2}\left[\mu_{4}-\mu_{2}^{2}\right]+\frac{1}{4}{\left[f^{\prime }\left(\mu \right)\right]}^{2}\mu_{2}+\frac{1}{4}f^{\prime }\left(\mu \right)f^{\prime \prime }\left(\mu \right)\mu_{3},$$

*where $\mu_{r}=E{\left(X-\mu \right)}^{r}<+\infty ,\;r=2,3,4$.*

**Proof.**  Making use of (2) and (3), then

$$\begin{array}{ccc} VJ(X) & = & \frac{1}{4}Var\left[f\left(X\right)\right]=\frac{1}{4}E\left[{\left(f\left(X\right)-E\left(f\left(X\right)\right)\right)}^{2}\right]\\ & \approx & \frac{1}{4}E{\left[f\left(\mu \right)+f^{\prime }\left(\mu \right)\left(X-\mu \right)+\frac{1}{2}f^{\prime \prime }\left(\mu \right){\left(X-\mu \right)}^{2}-E\left(f\left(X\right)\right)\right]}^{2}\\ & = & \frac{1}{4}E{\left[f\left(\mu \right)-E\left(f\left(X\right)\right)+f^{\prime }\left(\mu \right)\left(X-\mu \right)+\frac{1}{2}f^{\prime \prime }\left(\mu \right){\left(X-\mu \right)}^{2}\right]}^{2}\\ & \approx & \frac{1}{4}E{\left[-\frac{1}{2}f^{\prime \prime }\left(\mu \right)Var\left(X\right)+f^{\prime }\left(\mu \right)\left(X-\mu \right)+\frac{1}{2}f^{\prime \prime }\left(\mu \right){\left(X-\mu \right)}^{2}\right]}^{2}. \end{array}$$

Therefore, the stated result follows.    □

**Definition** **2.** *For any random variables X and Y with pdf’s f and g respectively, the maximal correlation of X and Y is defined by*

$$\begin{array}{c} \tilde{\rho }\left(X,Y\right)=\max\frac{E[(f(X)-E[f(X)])(g(Y)-E[g(Y)])]}{\sqrt{Var[f(X)]Var[g(Y)]}}=\max\frac{Cov(f(X),g(Y))}{4\sqrt{VJ(X)VJ(Y)}}. \end{array}$$

*Note that $0\leq \tilde{\rho }\left(X,Y\right)\leq 1$. Moreover, $\tilde{\rho }\left(X,Y\right)=0$ if, and only if, X and Y are independent. See Beigi and Goahri [13] for for more details.*

**Remark** **3.** *If X is a random variable with pdf f and Y=|X|, then $\tilde{\rho }\left(X,Y\right)=1$.*

Let (X,Y) denote the lifetimes of two components of a system with joint cdf F(x,y) and joint pdf f(x,y). It is possible to introduce the bivariate version of extropy, denoted by J(X,Y), in the following way:

$$J\left(X,Y\right)=\frac{1}{4}E\left[f\left(X,Y\right)\right]=\frac{1}{4}\int_{0}^{+\infty }\int_{0}^{+\infty }f^{2}\left(x,y\right)dxdy,$$

see Balakrishnan et al. [14] for further details. Hence, the bivariate VJ is given by

$$\begin{array}{c} VJ\left(X,Y\right):=VJ\left(f\right)=\frac{1}{16}Var\left[f\left(X,Y\right)\right]. \end{array}$$

In the case when *X* and *Y* are independent random variables, then

$$VJ\left(X,Y\right)=VJ\left(X\right)VJ\left(Y\right)+VJ\left(X\right)J^{2}\left(Y\right)+J^{2}\left(X\right)VJ\left(Y\right),$$

and, if in addition, *X* and *Y* are identically distributed, then

$$VJ\left(X,Y\right)=VJ^{2}\left(X\right)+2J^{2}\left(X\right)VJ\left(Y\right)=VJ\left(X\right)\left(VJ\left(X\right)+2J^{2}\left(X\right)\right).$$

For example, let $X,Y\overset{iid}{∼}N\left(0,1\right)$, then, by using Example 1 and $J\left(X\right)=-\frac{1}{4\sqrt{\pi }}$, then $VJ\left(X,Y\right)=\frac{{\left(2-\sqrt{3}\right)}^{2}}{768\pi^{2}}+\frac{2-\sqrt{3}}{128\pi^{2}\sqrt{3}}=\frac{1}{768\pi^{2}}$.

### 2.1. Residual and Past Varextropies

As mentioned in the introduction, several researchers have dedicated their attention to the study of extropy. Now, we recall the definitions of residual and past extropy. Let *X* be a non negative and absolutely continuous random variable, then $X_{t}=\left[X-t|X\geq t\right]$ is the residual lifetime with pdf $f_{t}\left(x\right)=f\left(x+t\right)/\bar{F}\left(t\right),\;x>0$ and $X_{\left[t\right]}=\left[X|X\leq t\right]$ is the past lifetime with pdf $f_{\left[t\right]}\left(x\right)=\frac{f(x)}{F(t)},\;0<x<t$. In analogy with the residual entropy, Qiu [10] defined the extropy for residual lifetime $X_{t}$, i.e., the residual extropy at time *t*, as

$$J\left(X_{t}\right)=-\frac{1}{2}\int_{0}^{+\infty }f_{X_{t}}^{2}\left(x\right)dx=-\frac{1}{2{\bar{F}}^{2}\left(t\right)}\int_{t}^{+\infty }f^{2}\left(x\right)dx.$$

About the past lifetime $X_{\left[t\right]}=\left(X|X\leq t\right)$, Krishnan et al. [15] and Kamari and Buono [16] studied the past extropy defined as

$$J\left(X_{\left[t\right]}\right)=-\frac{1}{2}\int_{0}^{+\infty }f_{X_{\left[t\right]}}^{2}\left(x\right)dx=-\frac{1}{2F^{2}\left(t\right)}\int_{0}^{t}f^{2}\left(x\right)dx.$$

The residual extropy and the past extropy can be seen as expectations. So, the residual and the past varextropies of *X* at time *t*, $VJ(X_{t})$ and $VJ(X_{\left[t\right]})$ are

$$\begin{array}{cc} VJ(X_{t}) & =\frac{1}{4}E\left[f_{X_{t}}^{2}\left(X_{t}\right)\right]-J^{2}\left(X_{t}\right),\\ VJ(X_{\left[t\right]}) & =\frac{1}{4}E\left[f_{X_{\left[t\right]}}^{2}\left(X_{\left[t\right]}\right)\right]-J^{2}\left(X_{\left[t\right]}\right). \end{array}$$

**Example** **2.**

*(i)*  *If X has an exponential distribution, then*
  $$VJ\left(X_{t}\right)=VJ\left(X_{\left[t\right]}\right)=VJ\left(X\right),$$
  *i.e., it is independent of the lifetime of the system.*
*(ii)*  *If X follows a power distribution with parameter α>1, then*
  $$\begin{array}{cc} \; & VJ\left(X_{t}\right)=\frac{\alpha^{3}}{4{\left(1-t^{\alpha }\right)}^{4}}\left[\frac{\left(1-t^{3\alpha -2}\right)\left(1-t^{\alpha }\right)}{3\alpha -2}-\frac{\alpha {\left(1-t^{2\alpha -1}\right)}^{2}}{{\left(2\alpha -1\right)}^{2}}\right],\\ \; & VJ\left(X_{\left[t\right]}\right)=\frac{\alpha^{3}{\left(\alpha -1\right)}^{2}}{4t^{2}\left(3\alpha -2\right){\left(2\alpha -1\right)}^{2}}. \end{array}$$

**Proposition** **5.** *If Y=aX+b, with X non-negative random variable, a>0 and b≥0, then*

$$\begin{array}{cc} VJ(Y_{t}) & =\frac{1}{a^{2}}VJ\left(X_{\frac{t-b}{a}}\right),\\ VJ(Y_{\left[t\right]}) & =\frac{1}{a^{2}}VJ\left(X_{\left[\frac{t-b}{a}\right]}\right). \end{array}$$

It is observed that the residual varextropy can be written as

$$VJ\left(X_{t}\right)=\frac{Var[f(X+t)]}{4{\bar{F}}^{2}\left(t\right)},$$

hence, for all t≥0, the derivative of the residual varextropy is

$$\frac{\partial }{\partial t}VJ\left(X_{t}\right)=VJ\left(X_{t}\right)\left[2\lambda_{X}\left(t\right)+\frac{\partial }{\partial t}\log\left(Var\left[f\left(X+t\right)\right]\right)\right],$$

where $\lambda_{X}\left(t\right)=\frac{f(t)}{\bar{F}\left(t\right)}$ is the hazard rate function. Solving the above differential equation leads to

$$VJ\left(X_{t}\right)=\exp\left[\int [2\lambda_{X}\left(t\right)+\frac{\partial }{\partial t}\log\left(Var\left[f\left(X+t\right)\right]\right)]dt+C\right],$$

where *C* is a constant.

### 2.2. Reliability Theory

Hereafter, we consider two non-negative random variables *X* and $X_{\theta }$ with cdf’s F(x) and $F^{*}\left(x\right)$, respectively. These variables satisfy the proportional hazard rate model (PHRM) with proportionality constant θ>0, if

$${\bar{F}}_{X_{\theta }}^{*}\left(x\right)={\left[\bar{F}\left(x\right)\right]}^{\theta },\;x>0.$$

For detail on PHRM and some properties of such a model associated with aging notions, see Gupta and Gupta [17].

**Proposition** **6.** *Let X be a non-negative absolutely continuous random variable with cdf F and pdf f. Then,*

(4) $$\begin{array}{c} VJ\left(X_{\theta }\right)=\frac{\theta^{3}}{4}E\left[f^{2}\left(F^{-1}\left(1-U\right)\right)U^{3(\theta -1)}\right]-\frac{\theta^{4}}{4}E^{2}\left[f\left(F^{-1}\left(1-U\right)\right)U^{2(\theta -1)}\right], \end{array}$$

*where U∼U(0,1).*

In reliability theory (n−k+1)-out-of *n* systems, k∈{1,…,n}, are important types of structures. If $X_{1},X_{2},\ldots ,X_{n}$ denote the independent lifetimes of the components of a (n−k+1) -out-of *n* system, then the lifetime of this system is equal to the order statistic $X_{k:n}$. Hence, in the following proposition, we obtain an analytical expression for $VJ(X_{k:n})$.

**Proposition** **7.** *Let $X_{1},X_{2},\ldots ,X_{n}$ be a random sample from an absolutely continuous cdf F(x), then*

$$\begin{array}{ccc} VJ(X_{k:n}) & = & \frac{B(3k-2,3(n-k)+1)}{4B^{3}\left(k,n-k+1\right)}E\left[f^{2}\left(F^{-1}\left(U_{1}\right)\right)\right]\\ & & -\frac{B^{2}\left(2k-1,2\left(n-k\right)+1\right)}{4B^{2}\left(k,n-k+1\right)}E^{2}\left[f(F^{-1}\left(U_{2}\right))\right], \end{array}$$

*where $U_{1}∼Beta\left(3k-2,3\left(n-k\right)+1\right)$ and $U_{2}∼Beta\left(2k-1,2\left(n-k\right)+1\right)$.*

**Remark** **4.** *Let $X_{1:n}=\min\left\{X_{1},X_{2},\ldots ,X_{n}\right\}$ and $X_{n:n}=\max\left\{X_{1},X_{2},\ldots ,X_{n}\right\}$ denote the lifetime of the series and parallel systems, respectively. Then,*

*(i)*  $VJ\left(X_{1:n}\right)=\frac{n^{3}}{4}E\left[f^{2}\left(F^{-1}\left(1-U\right)\right)U^{3(n-1)}\right]-\frac{n^{4}}{4}E^{2}\left[f\left(F^{-1}\left(1-U\right)\right)U^{2(n-1)}\right];$
*(ii)*  $VJ\left(X_{n:n}\right)=\frac{n^{3}}{4}E\left[f^{2}\left(F^{-1}\left(U\right)\right)U^{3(n-1)}\right]-\frac{n^{4}}{4}E^{2}\left[f\left(F^{-1}\left(U\right)\right)U^{2(n-1)}\right];$

*we note that (i) coincides with (4), since the series system is a particular case of PHRM with the choice of parameter θ=n.*

**Proposition** **8.** *Let $X_{1},X_{2},\ldots ,X_{n}$ be a random sample from continuous symmetric distribution F(x), then*

$$\begin{array}{c} VJ\left(X_{k:n}\right)=VJ\left(X_{n-k+1:n}\right). \end{array}$$

In the following, a few examples are given to illustrate the varextropy for order statistics $X_{k:n}$ from some distributions.

**Example** **3.**

*(i)*  *If X is uniformly distributed in [a,b], then*
  $$VJ\left(X_{k:n}\right)={\left(\frac{1}{b-a}\right)}^{2}\left(\frac{B(3k-2,3(n-k)+1)}{4B^{3}\left(k,n-k+1\right)}-\frac{B^{2}\left(2k-1,2\left(n-k\right)+1\right)}{4B^{2}\left(k,n-k+1\right)}\right);$$
*(ii)*  *If X has exponential distribution with parameter θ, then*
  $$VJ\left(X_{k:n}\right)=\theta^{2}\left(\frac{B(3k-2,3(n-k)+3)}{4B^{3}\left(k,n-k+1\right)}-\frac{B^{2}\left(2k-1,2\left(n-k\right)+2\right)}{4B^{2}\left(k,n-k+1\right)}\right);$$
*(iii)*  *If X has Pareto distribution with shape and scale parameters λ and β respectively, then*
  $$VJ\left(X_{k:n}\right)=\frac{\lambda^{2}}{\beta^{2}}\left(\frac{\beta (3k-2,3\left(n-k\right)+\frac{2}{\lambda }+3)}{4\beta^{3}\left(k,n-k+1\right)}-\frac{\beta^{2}\left(2k-1,2\left(n-k\right)+\frac{1}{\lambda }+3\right)}{4\beta^{2}\left(k,n-k+1\right)}\right).$$

Table 1 gives the numerical values of $VJ(X_{k:n})$ with n=10 for the standard uniform distribution. It can be observed that $VJ\left(X_{k:n}\right)=VJ\left(X_{n-k+1:n}\right)$, as stated in Proposition 8, and $VJ(X_{k:n})$ is increasing with respect to *k* for $k\geq \frac{n+1}{2}\;\left(\frac{n}{2}+1\right)$ when *n* is odd (even). Furthermore, it is decreasing with respect to *k* for $k\leq \frac{n+1}{2}\;\left(\frac{n}{2}\right)$. Therefore, the median of order statistics has a minimum varextropy. It should be noted that, when *n* is even, both of the middle random variables of order statistics, are the median.

In reliability tests, some products may fail under stress. In such experiments for getting the precise failure point, measurements may be made sequentially, and only values larger (or smaller) than all previous ones are recorded. Let $X_{1},X_{2},\ldots ,X_{n},\ldots ,$ be a sequence of iid random variables with cdf F(x) and pdf f(x). An observation $X_{j}$ is called an upper record (lower record) value if $X_{j}>\left(<\right)\;X_{i},\;\forall i<j$. In the following, we obtain varextropy measures for upper record values.

**Proposition** **9.** *Let $X_{U_{n}}$ be n-th upper record value with pdf $f_{n}\left(x\right)=\frac{1}{(n-1)!}{\left[-\log\left(1-F\left(x\right)\right)\right]}^{n-1}$f(x), then*

$$\begin{array}{ccc} VJ\left(X_{U_{n}}\right)=\frac{1}{4}Var\left[f\left(X_{U_{n}}\right)\right] & = & \frac{(3n-3)!}{4{\left[\left(n-1\right)!\right]}^{3}}E\left[f^{2}\left(F^{-1}\left(1-e^{-V}\right)\right)\right]\\ & - & \frac{{\left[\left(2n-2\right)!\right]}^{2}}{4{\left[\left(n-1\right)!\right]}^{4}}E^{2}\left[f\left(F^{-1}\left(1-e^{-W}\right)\right)\right],\;\;n>1, \end{array}$$

*where V∼Gamma(3n−2,1) and W∼Gamma(2n−1,1).*

### 2.3. The Discrete Case

In analogy with (2), the varextropy of a discrete random variable *X* taking values in the set $\left\{x_{i},\;i\in I\right\}$ is expressed as

$$VJ\left(X\right)=\frac{1}{4}\left[\sum_{i\in I}^{}P^{3}\left(X=x_{i}\right)-{\left(\sum_{i\in I}^{}P^{2}\left(X=x_{i}\right)\right)}^{2}\right].$$

**Example** **4.** *Let Y be a Bernoulli random variable having distribution P(Y=0)=1−θ, P(Y=1)=θ, with 0≤θ≤1, then the varextropy is given by*

$$VJ\left(Y\right)=0.25\left[{\left(1-\theta \right)}^{3}+\theta^{3}-{\left(1-\theta \right)}^{4}-\theta^{4}-2\theta^{2}{\left(1-\theta \right)}^{2}\right].$$

**Figure 1:**
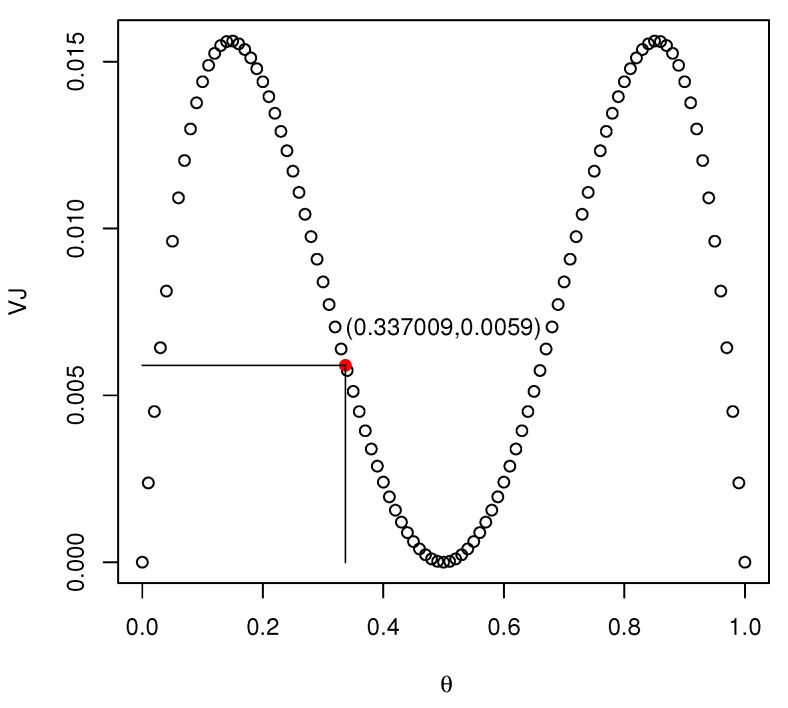
The values of VJ(Y) for Bernoulli distribution.

*Figure 1 shows the values of VJ(Y) as θ varies in [0,1], it can be seen that 0≤VJ(Y)≤0.0156. Note that for $\theta =\theta^{*}=0.337009$, H(Y)=J(Y)=0.639032 and VJ(Y)=0.0059.*

**Example** **5.** *Let X be a discrete random variable such that, for a fixed h>0,*

P(X=h)=p,P(X=0)=1−p−q,P(X=−h)=q,

*with 0≤q≤1−p≤1. We have*

$$VJ\left(X\right)=0.25\left[p^{3}+{\left(1-p-q\right)}^{3}+q^{3}-{\left(p^{2}+{\left(1-p-q\right)}^{2}+q^{2}\right)}^{2}\right].$$

*Now, by using the function* *fzero* *of MATLAB, it is found that if p=q=0.1508, then J(X)=0.639032 and VJ(X)=0.0158. Hence, with this choice of parameters, the considered random variable has the same entropy as the one considered in Example 4 with $\theta =\theta^{*}$, but the varextropy of X is larger. This implies that the coding procedure is more reliable for sequences generated by the random variable Y considered in Example 4.*

## 3. General Results on Conditional Varextropy

Henceforward, we investigate some results on the conditional VJ of a random phenomenon. Assume that *X* is a random variable defined on the probability space (Ω,F,P) and such that E|X|<+∞. The conditional variance of *X* given sub σ-field G is denoted by Var(X|G), where G⊂F. Here, the definition of the conditional VJ of *X* is given and some of its properties are discussed.

**Definition** **3.** *Let X be a non-negative random variable with pdf f such that $E(f^{2}\left(X\right))<+\infty$. Then, for a given σ-field F, the conditional VJ is defined as follows:*

$$\begin{array}{c} VJ\left(X|F\right)=\frac{1}{4}Var\left[f\left(X\right)|F\right]=\frac{1}{4}E\left[{\left(f\left(X\right)-E\left(f\left(X\right)|F\right)\right)}^{2}|F\right]. \end{array}$$

In the following proposition, the varextropy version of the law of total variance is given.

**Proposition** **10.** *Suppose that X is a random variable with pdf f, then*

$$\begin{array}{ccc} VJ\left(X\right)=\frac{1}{4}Var\left[f\left(X\right)\right] & = & \frac{1}{4}E\left[Var\left[f\left(X\right)|F\right]\right]+\frac{1}{4}Var\left[E\left[f\left(X\right)|F\right]\right]\\ & = & E\left(VJ\left(X|F\right)\right)+\frac{1}{4}Var\left[E\left[f\left(X\right)|F\right]\right]. \end{array}$$

*It is clear that VJ(X)≥E(VJ(X|F)).*

**Lemma** **1.** *Let X be a random variable with cdf F and support (0,+∞). If F={∅,Ω}, then VJ(X|F)=VJ(X).*

**Proposition** **11.** *Let $E(f^{2}\left(X\right))<+\infty$. Then, for σ-fields G⊂F*

(5) $$\begin{array}{c} E(VJ(X|G))\leq E(VJ(X|F)). \end{array}$$

**Theorem** **2.** *Let $E(f^{2}\left(X\right))<+\infty$ and let F be a σ- field. Then E(VJ(X|F))=0 if, and only if, f(x)=c, where c is a non negative constant, and X is F-measurable.*

**Proof.**  If we assume that E(VJ(X|F))=0, then VJ(X|F)=0. Recalling the definition of VJ(X|F), then Var[f(X)|F]=0. So, *f* is a constant function and *X* is F-measurable.

Let us suppose that f(x)=c, where c>0 is a constant, and *X* is F-measurable. Again, by using Definition 3, we have Var[f(X)|F]=0, so the result follows.  □

From the Markov property of the lifetime random variables X,Y and *Z*, we have the following lemma.

**Lemma** **2.** *If X→Y→Z is a Markov chain, then*

*(i)*  VJ(Z|Y,X)=VJ(Z|Y).
*(ii)*  E[VJ(Z|Y)]≤E[VJ(Z|X)].

**Proof.** 

(i) By using the Markov property and definition of VJ(Z|Y,X), the result follows.
(ii) Let G=σ(X) and F=σ(X,Y), then from (5), we have
  $$\begin{array}{ccc} E[VJ(Z|X)] & \geq & E(E[VJ(Z|X,Y)|X])=E[VJ(Z|X,Y)]=E[VJ(Z|Y)], \end{array}$$
  and the result follows.

□

**Remark** **5.** *Let (X,Y) denote the lifetimes of two components of a system with joint density function f(x,y). Another measure of correlation is the maximal correlation ribbon (MC ribbon) defined in Beigi and Gohari [18]. The MC ribbon is equal to the set of $\left(\lambda_{1},\lambda_{2}\right)\in {\left[0,1\right]}^{2}$ such that we have*

$$\begin{array}{c} VJ\left(f\right)\geq \lambda_{1}VJ\left(E\left[f|X\right]\right)+\lambda_{2}VJ\left(E\left[f|Y\right]\right). \end{array}$$

## 4. Stochastic Comparisons

Before proceeding to give the results of this section, we need the following definitions on stochastic orderings between random variables. For more details on these concepts, one can see Shaked and Shanthikumar [19].

**Definition** **4.** *Suppose that X and Y are two random variables with density functions f and g and survival functions $\bar{F}\left(x\right)=1-F\left(x\right)$ and $\bar{G}\left(x\right)=1-G\left(x\right)$, respectively. Then,*

*1*  *X is smaller than Y in the stochastic ordering, denoted by $X\overset{st}{\leq }Y$, if $\bar{F}\left(t\right)\leq \bar{G}\left(t\right)$ for all t;*
*2*  *X is smaller than Y in the likelihood ratio ordering, denoted by $X\overset{lr}{\leq }Y,$ if $\frac{g(t)}{f(t)}$ is increasing in t>0;*
*3*  *X is smaller than Y in the hazard rate order, denoted by $X\overset{hr}{\leq }Y$, if $\lambda_{X}\left(x\right)\geq \lambda_{Y}\left(x\right)$ for all x;*
*4*  *X is smaller than Y in the dispersive order, denoted by $X\overset{disp}{\leq }Y$, if $f\left(F^{-1}\left(u\right)\right)\geq g\left(G^{-1}\left(u\right)\right)$ for all u∈(0,1), where $F^{-1}$ and $G^{-1}$ are right continuous inverses of F and G, respectively;*
*5*  *X is said to have decreasing failure rate (DFR) if $\lambda_{X}\left(x\right)$ is decreasing in x;*
*6*  *X is smaller than Y in the convex transform order, denoted by $X\overset{c}{\leq }Y$, if $G^{-1}\left(F\left(x\right)\right)$ is a convex function on the support of X;*
*7*  *X is smaller than Y in the star order, denoted by $X\overset{*}{\leq }Y$, if $\frac{G^{-1}F\left(x\right)}{x}$ is increasing in x≥0;*
*8*  *X is smaller than Y in the superadditive order, denoted by $X\overset{su}{\leq }Y$, if $G^{-1}\left(F\left(t+u\right)\right)\geq G^{-1}\left(F\left(t\right)\right)+G^{-1}\left(F\left(u\right)\right)$ for t≥0,u≥0.*

In the following, we introduce a new stochastic order based on the varextropy.

**Definition** **5.** *The random variable X is said to be smaller than Y in the varextropy order, denoted by $X\overset{VJ}{\leq }Y$, if VJ(X)≤VJ(Y).*

In the following example, we get some comparisons about the varextropy order based on the results given in Example 1.

**Example** **6.**

*(i)*  *If X∼Laplace(0,1) and Y∼Exp(1), then we have $X\overset{VJ}{\leq }Y$, since $VJ\left(X\right)=\frac{1}{192}$ and $VJ\left(Y\right)=\frac{1}{48}$;*
*(ii)*  *If X∼Weibull(1,2) and Y∼Weibull(1,1), then we have $X\overset{VJ}{\leq }Y$, since VJ(X)=0.0129 and VJ(Y)=0.02;*
*(iii)*  *If $X∼Exp(\lambda_{1})$ and $Y∼Exp(\lambda_{2})$ with $\lambda_{1}\leq \lambda_{2}$, then $X\overset{VJ}{\leq }Y$;*
*(iv)*  *If $X∼N(\mu_{1},\sigma_{1}^{2})$ and $Y∼N(\mu_{2},\sigma_{2}^{2})$ with $\sigma_{2}\leq \sigma_{1}$, then $X\overset{VJ}{\leq }Y$.*

**Remark** **6.** *$X\overset{VJ}{\leq }Y\Leftrightarrow \left|X\right|\overset{VJ}{\leq }\left|Y\right|$, where the equivalence follows from Remark 1.*

Since the varextropy is defined as the variance of the pdf multiplied by a constant, it is known that the uniform distribution is the only one for which the varextropy vanishes. Then, the following result is obtained.

**Proposition** **12.** *If X∼U(a,b), then $X\overset{VJ}{\leq }Y$ for any continuous random variable Y.*

**Proposition** **13.** *Let $X_{k:n}$ be kth order statistic of standard uniform distribution, then*

*(i)*  *$X_{k:n}\overset{VJ}{\leq }X_{1:n}$ and $X_{k:n}\overset{VJ}{\leq }X_{n:n}$ for all 1≤k≤n.*
*(ii)*  *when n is even, we have $X_{\frac{n}{2}:n}\overset{VJ}{\leq }X_{k:n}$ for all 1≤k≤n.*
*(iii)*  *when n is odd, we have $X_{\frac{n+1}{2}:n}\overset{VJ}{\leq }X_{k:n}$ for all 1≤k≤n.*

**Remark** **7** (Chernoff [20]). *Let X be a random variable with standard normal distribution. If h is absolutely continuous and h(X) has finite variance, then*

(6) $$Var\left(h\left(X\right)\right)\leq E\left\{{\left[h^{\prime }\left(X\right)\right]}^{2}\right\}.$$

From Remark 7, the following result is obtained.

**Corollary** **1.** *If X is a standard normal random variable and $h\left(x\right)=\frac{1}{\sqrt{2\pi }}e^{\frac{-x^{2}}{2}}$, then*

$$VJ\left(X\right)\leq \frac{1}{8\pi \sqrt{27}}.$$

In the following, a lower bound for VJ(X) based on Chebyshev inequality is given.

**Corollary** **2.** *Let X be a random variable with pdf f(x), then*

VJ(X)≥P(|f(X)+2J(X)|≥2).

Finally, the following results from Shaked and Shanthikumar [19] are provided.

**Proposition** **14.** *If X and Y are two random variables such that $X\overset{disp}{\leq }Y$, then $X\overset{VJ}{\geq }Y$.*

**Example** **7.** *Let $F_{X}\left(t\right)=1-\exp\left(-2t\right),\;t>0$ and $G_{Y}\left(t\right)=1-\exp\left(-t\right),\;t>0$. Then $X\overset{disp}{\leq }Y$ implies $X\overset{VJ}{\geq }Y$.*

**Corollary** **3.** *If $X\overset{hr}{\leq }Y$, and X or Y is DFR, then $X\overset{VJ}{\geq }Y$.*

**Proof.**  If $X\overset{hr}{\leq }Y$, and *X* or *Y* is DFR, then $X\overset{disp}{\leq }Y$, due to Bagai and Kochar [21]. Thus, from Proposition 14, the result follows.  □

**Corollary** **4.** *If $X\overset{su}{\leq }Y\;\left(X\overset{*}{\leq }Y\;or\;X\overset{c}{\leq }Y\right)$ and f(0)≥g(0)>0. Then $X\overset{VJ}{\geq }Y$.*

**Proof.**  If $X\overset{su}{\leq }Y\;\left(X\overset{*}{\leq }Y\;or\;X\overset{c}{\leq }Y\right)$ and f(0)≥g(0)>0, then $X\overset{disp}{\leq }Y$, due to Ahmed et al. [22]. So, from Proposition 14, the result follows.  □

**Corollary** **5.** *Suppose that $X_{k:n}$ and $Y_{k:n}$ are the kth order statistics of two continuous random variables X and Y, respectively. If $X\overset{disp}{\leq }Y$, then $X_{k:n}\overset{VJ}{\geq }Y_{k:n}$.*

**Proof.**  The proof follows by Theorem 3.B.26 of [19].  □

**Corollary** **6.** *If $X\overset{st}{\leq }Y\;\left(X\overset{lr}{\leq }Y\right)$, then $X\overset{VJ}{\geq }Y$.*

**Corollary** **7.** *Let X be a non negative random variable having a DFR distribution. If $X_{k:n}\overset{lr}{\leq }X$, then $X_{k:n}\overset{VJ}{\geq }X$.*

**Corollary** **8.** *Let X be a non negative random variable having a DFR distribution. Then, $X_{1:n}\overset{VJ}{\geq }X$ and $X_{n:n}\overset{VJ}{\leq }X$.*

## 5. Conclusions

In this paper, some properties of VJ were obtained. This measure can be applied for measuring the information volatility contained in the associated residual and past lifetimes. Some of its properties based on order statistics, record values and proportional hazard rate models were considered. Moreover, using Taylor series, the approximate formula for VJ(X) was proposed. Finally, the conditional VJ of a random phenomenon was discussed and a new stochastic order, named varextropy ordering, was introduced. To continue future works, we list some properties and advantages of varextropy and its extends, to highlight the rationality and value of the new method.

(1) VJ of a uniform random variable as well as VH are both equal to zero, see Example 1.
(2) The new introduced varextropy measure is more flexible than the varentropy, in the sense that the latter is free of the model parameters in some cases, see Example 1.
(3) In this case of normal distribution, the varextropy only depends on the scale parameter $\sigma^{2}$, see Example 1.
(4) For symmetric distributions, the VJ is unchanged under symmetry, see Proposition 3.
(5) VJ of half normal can be easily obtained via VJ of normal distribution, see Remark 1.
(6) VJ can be approximated using Taylor series, for further details see Theorem 1.
(7) VJ is invariant under translations, for further details, see Proposition 4.
(8) The residual VJ of an exponential distribution is independent of lifetime model, more specific explanation can be seen in Example 2.
(9) VJ of the PHRM can be obtained from the original model properties, see Proposition 6.
(10) For symmetric distributions, VJ of *k*-th order statistic is equal to VJ of (n−k+1)-th order statistic from a sample of size *n*, for further details see Proposition 8.
(11) The median of order statistics has a minimum VJ, more specific explanation can be seen in Section 2.2.
(12) VJ of a random variable *X* is bigger than that of the expected value of conditional VJ of *X*, see Proposition 10.
(13) If X→Y→Z is a Markov chain, then VJ(Z|Y,X)=VJ(Z|Y), for further details see Lemma 2.
(14) For the one-parameter exponential distribution, when the value of parameter increases then the exponential distribution increases in varextropy order, see Example 6.
(15) For the normal distribution, when the value of scale parameter increases then the normal distribution decreases in varextropy order independently of location parameter, see Example 6.
(16) If *X* is smaller than *Y* in varextropy order then the result also holds for absolute value of *X* and *Y* and vice versa, see Remark 6.
(17) Based on varextropy order, every continuous random variable is bigger than the uniform distribution, for further details, see Proposition 12.

## Figures and Tables

**Table 1:** The values of $VJ(X_{k:n})$ for the standard uniform distribution.

*k*	1	2	3	4	5
$VJ(X_{k:n})$	8.859319	2.225035	1.407279	1.129121	1.027418
*k*	6	7	8	9	10
$VJ(X_{k:n})$	1.027418	1.129121	1.407279	2.225035	8.859319

## Abbreviations

The following abbreviations are used in this manuscript:

cdf	Cumulative distribution function
pdf	Probability density function
VJ	Varextropy
